# Transient activation of retinopathy of prematurity secondary to erythrocyte suspension transfusion

**DOI:** 10.22336/rjo.2024.11

**Published:** 2024

**Authors:** Alparslan Şahin

**Affiliations:** * Department of Ophthalmology, Bower Hospital, Yenisehir, Diyarbakir, Turkey

**Keywords:** erythrocyte suspension, plus sign, retinopathy of prematurity, transfusion, transient activation

## Abstract

Retinopathy of prematurity (ROP) is a serious retinal vascular disorder that needs prompt diagnosis, and treatment to prevent undesired visual outcomes. Due to its shorter period of disease progression, it is important to be hasty in treating ROP. Erythrocyte suspension (ES) aggravates the progression of ROP. However, this progression may be transient as in the present case reports. This case report aimed to present two cases that developed type 1 ROP after erythrocyte suspension transfusion. Clinical findings of the patients were resolved within a few days without any intervention. Premature infants receiving ES treatment can be observed for 24-48 hours, and the treatment can be planned after determining the persistence of the plus sign.

**Abbreviations:** ES = Erythrocyte suspension, ROP = Retinopathy of prematurity, NICU = neonatal intensive care unit

## Introduction

Retinopathy of prematurity (ROP) is a serious retinal vascular disorder that may cause blindness. Several risk factors are defined for the progression of ROP. Erythrocyte suspension (ES) transfusion is a well-defined risk factor for ROP progression. Ninety percent of premature infants will get at least one ES transfusion [**1**]. Zhu et al. reported that ES transfusion is an independent risk factor for ROP progression, particularly in extremely preterm infants [**2**]. It has been reported that the incidence of ROP was 1.68 times higher in the ES transfusion group than in those of the non-transfused group of premature infants [**3**].

Prompt diagnosis and treatment are important to prevent devastating visual outcomes. Although it is necessary to be hasty in treating the disease, in some cases the delay in treatment may result in favor of the patient, as in the two case reports mentioned below. This report presented two cases with the diagnosis of type 1 ROP after ES transfusion that resolved within a few days after diagnosis without any surgical intervention. 

## Case reports

*Case 1*

**Fig. 1:**
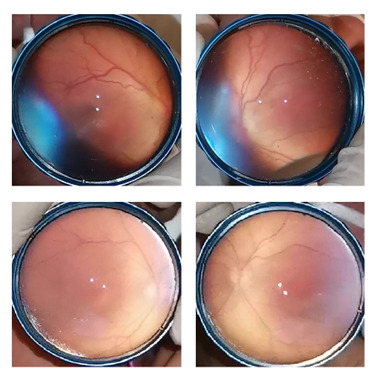
The fundus images of case 1. Note the plus sign just a day after ES transfusion (Top). Two days after this examination the plus sign recovered without any intervention (Bottom)

A 26-week-old infant with a birthweight of 790 grams was hospitalized in the neonatal intensive care unit (NICU). The first ocular examination of the patient was performed on the 28th day of age. It revealed bilateral mild vitreous haze, but no plus disease, and zone I immature retina without any stage of ROP. Consequent two examinations revealed the same findings other than resolving vitreous haze. Unfortunately, the fourth examination revealed a significant plus disease with stage 1 disease in zone I. Hence, bilateral intravitreal injection of bevacizumab was recommended. However, the treatment of the patient was delayed because of waiting for the legal guardians’ consent and lack of drug availability. Two days after diagnosis, a bilateral injection of bevacizumab was scheduled. Before injection, the retinal examination was performed. It was observed that the bilateral plus sign significantly disappeared with stable retinal disease as stage 1 in zone I. Because of the detailed examination of the patient’s medical history, it was observed that the patient was given an ES transfusion a day before the appearance of the plus disease. Treatment was cancelled. Consequently, the follow-up examinations did not show progression of ROP. Retinal vascularization was completed without any intervention (**Fig. 1**).

*Case 2*

**Fig. 2:**
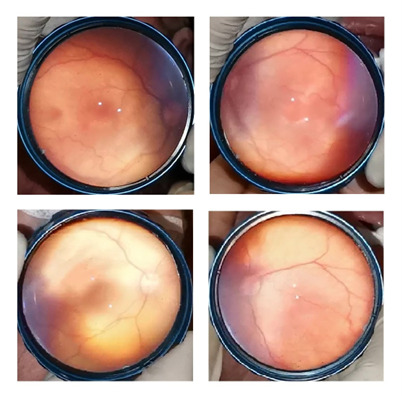
The fundus images of case 2. The plus sign 2 days after ES transfusion (Top). Plus sign disappeared two days after the previous examination (Bottom)

A 27-week-old infant with a birthweight of 1000 grams was followed in the NICU. The first two examinations did not reveal type 1 ROP (bilateral zone I immature retinal vascularization without plus sign). The third examination revealed bilateral zone I stage 1 disease with plus disease. The patient had received an ES transfusion two days before this examination. Hence, a closer follow-up was recommended. Two days later, the examination revealed complete recovery of plus sign with zone I stage 1 disease. The patient was followed up closely. The ROP was regressed and complete retinal vascularization was observed without any complication (**Fig. 2**).

## Discussion

There are well-defined risk factors for the development of ROP [**4**]. Transfusion of ES is one of the reported risk factors [**2**]. ES transfusion is usually needed if the infants’ vital hemodynamic findings are impaired. Almost all extreme premature infants will get at least one ES transfusion [**5**]. ES transfusion primarily affects ROP through two mechanisms [**6**]. Because of their immature antioxidant system and high transferrin saturation, preterm newborns are particularly susceptible to the oxidative stress induced by free iron overload. Secondly, HbA, adult hemoglobin, has a lower affinity for oxygen than that of HbF. Therefore, it may lead to more release of oxygen to the tissues due to its lower affinity. 

The present case reports indicated that ES transfusion aggravated the plus sign that needs treatment in infants with ROP. Besides, waiting for a few days after ES transfusion might prevent unnecessary treatments in these infants. It should be kept in mind that ES transfusion may cause transient acceleration of the clinical findings of ROP due to ES transfusion, especially plus sign, and the infants should be followed closely. It can be interpreted that a few days after blood transfusion, the plus sign may improve as these babies stabilize hemodynamically.

## Conclusion

In the case of ROP progression, factors that worsen clinical findings should be questioned before treatment. Patients receiving ES treatment can be observed for 24-48 hours, and the treatment can be planned after determining the persistence of the plus sign. However, close follow-up is warranted after ES transfusion.

**Conflict of Interest Statement**

The author declares no conflict of interest. 

**Informed Consent and Human and Animal Rights Statement**

Informed consent has been obtained from all individuals included in this study. Minor patient consent to publish the case and images were gathered. 

**Authorization for the use of human subjects**

Ethical approval: The research related to human use complies with all the relevant national regulations and institutional policies, is by the tenets of the Helsinki Declaration, and has been approved by the Ethical Committee of Bower Hospital, Diyarbakir, Turkey (2023/0901, 15.09.2023).

**Acknowledgments**

None.

**Sources of Funding**

The author received no financial support for the research, authorship, and/or publication of this article. 

**Disclosures**

None.
